# Radiomics based on ^18^F-FDG PET/CT for prediction of pathological complete response to neoadjuvant therapy in non-small cell lung cancer

**DOI:** 10.3389/fonc.2024.1425837

**Published:** 2024-07-26

**Authors:** Jianjing Liu, Chunxiao Sui, Haiman Bian, Yue Li, Ziyang Wang, Jie Fu, Lisha Qi, Kun Chen, Wengui Xu, Xiaofeng Li

**Affiliations:** ^1^ Department of Molecular Imaging and Nuclear Medicine, Tianjin Medical University Cancer Institute and Hospital, Tianjin, China; ^2^ National Clinical Research Center for Cancer, Tianjin’s Clinical Research Center for Cancer, Key Laboratory of Cancer Prevention and Therapy, Tianjin, China; ^3^ Department of Molecular Imaging and Nuclear Medicine, Tianjin Cancer Hospital Airport Hospital, Tianjin, China; ^4^ Department of Radiology, Tianjin Medical University Cancer Institute and Hospital, Tianjin, China; ^5^ Department of Lung Cancer, Tianjin Medical University Cancer Institute and Hospital, Tianjin, China; ^6^ Department of Pathology, Tianjin Medical University Cancer Institute and Hospital, Tianjin, China

**Keywords:** ^18^F-FDG PET/CT, radiomics, NSCLC, neoadjuvant therapy, pathological complete response

## Abstract

**Purpose:**

This study aimed to establish and evaluate the value of integrated models involving ^18^F-FDG PET/CT-based radiomics and clinicopathological information in the prediction of pathological complete response (pCR) to neoadjuvant therapy (NAT) for non-small cell lung cancer (NSCLC).

**Methods:**

A total of 106 eligible NSCLC patients were included in the study. After volume of interest (VOI) segmentation, 2,016 PET-based and 2,016 CT-based radiomic features were extracted. To select an optimal machine learning model, a total of 25 models were constructed based on five sets of machine learning classifiers combined with five sets of predictive feature resources, including PET-based alone radiomics, CT-based alone radiomics, PET/CT-based radiomics, clinicopathological features, and PET/CT-based radiomics integrated with clinicopathological features. Area under the curves (AUCs) of receiver operator characteristic (ROC) curves were used as the main outcome to assess the model performance.

**Results:**

The hybrid PET/CT-derived radiomic model outperformed PET-alone and CT-alone radiomic models in the prediction of pCR to NAT. Moreover, addition of clinicopathological information further enhanced the predictive performance of PET/CT-derived radiomic model. Ultimately, the support vector machine (SVM)-based PET/CT radiomics combined clinicopathological information presented an optimal predictive efficacy with an AUC of 0.925 (95% CI 0.869–0.981) in the training cohort and an AUC of 0.863 (95% CI 0.740–0.985) in the test cohort. The developed nomogram involving radiomics and pathological type was suggested as a convenient tool to enable clinical application.

**Conclusions:**

The ^18^F-FDG PET/CT-based SVM radiomics integrated with clinicopathological information was an optimal model to non-invasively predict pCR to NAC for NSCLC.

## 1 Introduction

Lung cancer is a significant contributor to cancer-related mortality globally, with non-small cell lung cancer (NSCLC) comprising approximately 85% of all lung cancer cases (1–3). Despite the advancements in the treatment options for metastatic NSCLC, progress in the early-stage setting is limited (4). Driven by the necessity to enhance survival outcomes, renewed interest is emerging in exploring neoadjuvant strategies for early-stage and locally advanced NSCLC (5, 6).

Neoadjuvant therapy (NAT) encompasses various systemic treatment modalities administered prior to surgery, such as neoadjuvant chemotherapy, radiotherapy, targeted therapy, and immunotherapy (7). These therapies aim to reduce tumor burden, improve surgical outcomes, and ultimately enhance long-term survival rates for cancer patients (8). In 2020, the International Association for the Study of Lung Cancer (IASLC) released a recommendation for the pathological evaluation of neoadjuvant therapy, which introduced a standardized definition for major pathological response (MPR) as less than or equal to 10% viable tumor, whereas pathological complete response (pCR) indicated the absence of viable tumor (5). On the one hand, by virtue of the pathological response evaluation to neoadjuvant therapy, tumor sensitivity to systemic therapy is assessed at an early stage, which serves as a guide in determining the appropriate postoperative treatment strategy (9). On the other hand, previous clinical studies also proved that the pathological response to neoadjuvant treatment was a strong predictor for both disease-free survival and overall survival (10). Therefore, a noninvasive and reliable approach was in urgent need to predict the pathologic response to neoadjuvant therapy before treatment, which was beneficial to select potentially responsive NSCLC patients to administrate neoadjuvant therapy and maximize the therapeutic efficacy.

Computed tomography (CT), contrast-enhanced CT, and ^18^F-fluorodeoxyglucose positron emission tomography/computed tomography (^18^F-FDG PET/CT) are now commonly used imaging modalities in the clinical management of patients with lung cancer (11, 12). Particularly, PET/CT, as a hybrid imaging method, which is able to simultaneously provide metabolic information and anatomical details, is widely used in almost every aspect of clinical practice, including diagnosis, staging, treatment evaluation, and survival prognostication (13, 14). Consistently, several traditional metabolic parameters derived from PET images, such as maximum standardized uptake value (SUVmax), SUVmean, SUVpeak, metabolic tumor volume (MTV), and total lesion glycolysis (TLG), were previously identified as potential biomarkers in molecular subtype classification, pathological patterns determination, and outcome prediction for patients with NSCLC (15–18). However, as semi-quantitative parameters, especially for single-pixel value SUV, those commonly used conventional PET metabolic parameters were not able to reflect the complex heterogeneity existed in the images. More advanced methodology with enhanced predictive capability is expected to improve the prospect of NSCLC.

With the enormous improvement in the computing techniques in the era of big data, artificial intelligence, such as radiomics and machine learning, is increasingly becoming prevalent in the field of medical imaging (11, 19). In radiomics, a high throughput of features, which reflect the intra-tumor and inter-tumor heterogeneity are extracted, and a subset of informative radiomic features are finally selected after using a series of mathematical algorithms (20–22). In the end, multiple types of machine learning models are established and used as classifiers (23, 24). It is worth noting that these radiomic features are able to provide comprehensive heterogeneity information, which are usually not captured by the naked eyes. Though previous studies reported the roles of radiomics and machine learning based on CT in predicting pCR to NAT for NSCLC (6, 25, 26), few studies regarding PET/CT-derived radiomics were currently available.

In the present investigation, a total of 25 machine learning models, which involved five different combinations of predictive feature sources (PET-based alone radiomics, CT-based alone radiomics, PET/CT-based radiomics, clinicopathological features, and PET/CT radiomics integrated with clinicopathological features) and five different machine learning classifiers, were established to select the optimal model for predicting pCR to NAT in NSCLC. Furthermore, a nomogram with a visually straightforward representation was also constructed to detect the potential application of the developed machine learning models in clinical practice. This established machine learning model was potentially predictive of pCR to NAT in NSCLC, which provided a non-invasive approach to optimize the efficacy of NAT and improve the personalized treatment for NSCLC.

## 2 Materials and methods

### 2.1 Study population

A total of 188 NSCLC patients who underwent PET/CT imaging prior to NAT and surgical resection from June, 2020 to July, 2022 were enrolled in the retrospective study according to inclusion and exclusion criteria (**Supplementary Materials**). Ultimately, 106 NSCLC patients were included in the analysis, which were randomly divided into a training cohort (n = 74) and a testing cohort (n = 32) with a ratio of 7:3. This retrospective study was approved by the Ethics Review Committee of Tianjin Medical University Cancer Institute and Hospital, and written informed consent was waived. All procedures performed on human participants were conducted in compliance with the declaration of Helsinki and relevant ethical guidelines.

### 2.2 NAT regimen and pathological assessment

All the included patients underwent three to four cycles of platinum-based neoadjuvant chemotherapy, some of which also received concurrent immunotherapy. Then, surgery was conducted on all patients within 4–6 weeks after NAT. The pathological response to NAT was evaluated based on biopsy after resection by two pathologists with over 10 years of experience. pCR was defined as absence of residual tumor in histopathological section after resection (27).

### 2.3 Image acquisition and calculation of conventional PET metabolic parameters

Before imaging, NSCLC patients were informed to fast for at least 6 h and maintain their blood glucose levels below 140 mg/dl. Then, each patient received an intravenous injection of 3.7–4.44 MBq/kg (0.1–0.12 mCi/kg) of ^18^F-FDG. After resting for approximately 60 min, the acquisition of images was carried out using the GE Discovery Elite PET/CT scanner (GE Medical Systems). A low-dose CT scan (helical pitch 0.75:1, 3.75-mm slice thickness, 120 kV and 50–80 mAs) was first performed to provide anatomical correlation and for attenuation correction purpose. Then, a PET scan, consisting of eight-bed positions with each bed position requiring a 2-min duration with increments of 16.2 cm (3D mode), was followed from the top of the skull to the distal femur. All PET/CT images were independently reviewed by two experienced experts specialized in PET/CT imaging, and any disagreement in the interpretation was resolved by consensus. To determine the volume of interest (VOI), a commercial software (PET VCAR; GE Healthcare, USA) on GE Advantage Workstation 4.6 (AW 4.6) was employed by applying an isocontour threshold of 41% of the maximum SUV (SUVmax) method (28). Within the VOI, calculations of SUVmax, SUVmean, and SUVpeak were automatically performed. MTV was defined as a volumetric measurement of a lesion exhibiting significantly high ^18^F-FDG uptake (29). TLG was another volumetric index that was calculated by multiplying MTV with SUVmean.

### 2.4 Image segmentation and feature extraction

Semi-automatic segmentation of VOI was performed on CT images and PET images using 3D Slicer (version: 4.11.20210226) software by two nuclear medicine physicians with more than 5-year experiences specialized in PET/CT imaging. Before feature extraction, the spacing of PET and CT images and their corresponding VOIs were resampled to 1 × 1 × 1 mm³. A total of 4,032 radiomic features were extracted for each of the included NSCLC patients using the Pyradiomics module in Python 3.7.0, including a set of 2,016 CT-based alone and 2,016 PET-based alone radiomic features. To normalize the data into a standardized intensity range, we employed Z-score normalization for each radiomic feature.

### 2.5 Feature selection

For radiomic feature selection, the interclass correlation coefficient (ICC) test was first performed to assess the intra-observer and inter-observer repeatability in radiomic feature extraction. Radiomic features with ICC ≥0.75 were indicative of good reproducibility and reliability (30), whereas features with ICC <0.75 were excluded from further analysis. Second, a Mann–Whitney U test was used to select features highly related to pathological response to NAT with a significance level of 0.05 (p < 0.05). Then, Pearson’s rank correlation analysis was conducted to eliminate or avoid feature redundancy. Features with Pearson’s correlation coefficients above 0.90 were potentially highly related, in which one of the paired two features with a lower AUC was excluded. Furthermore, Minimum Redundancy Maximum Relevance (MRMR) was also implemented to further select the most significant and independent features. In the end, the least absolute shrinkage and selection operator (LASSO) was employed to select features for constructing the LASSO equation and calculating the corresponding feature weights. By adjusting the regularization weight λ, LASSO effectively reduced the magnitude of regression coefficients toward zero and eliminated the coefficients of irrelevant features by setting them precisely to zero. To determine the optimal λ, 10-fold cross-validation with minimum criteria was used. Nonzero coefficient features were selected and fitted into the regression model forming a radiomics signature. A radiomic score (Rad_Score) was then computed for each patient by combining the retained features linearly weighted by their respective model coefficients. Three distinct radiomic models were developed depending on the source of the extracted radiomic features. Rad_CT model was a radiomic model based on CT-derived alone features, and Rad_PET model was a radiomic model based on PET-derived alone features. For Rad_PET/CT model, both CT- and PET-based radiomic features were included to select a subset of predictive radiomic features to establish radiomic model. For feature selection of clinicopathological information, we followed a two-step procedure. First, univariate logistic regression analysis was conducted to identify significant features with a p-value <0.05. Then, the stepwise multivariate logistic regression analysis was performed on the aforementioned significant features to determine the independent indicator with a p-value <0.05, which were used as the predictive clinicopathological parameters to establish machine learning models for prediction of pCR to NAT for NSCLC.

### 2.6 Machine learning model construction

The imbalanced data between pCR and non-pCR groups (35:71) was corrected using synthetic minority over-sampling technique (SMOTE), in which the k-nearest neighbor algorithm was utilized to oversample the minority sample until achieving an equal number of cases in each group. After LASSO regression, the multiple sets of selected features were incorporated with five different types of machine learning classifiers, including Logistic Regression (LR), support vector machine (SVM), K-Nearest Neighbors (KNN), Light Gradient Boosting Machine (LightGBM), and NaiveBayes (NB), to construct corresponding machine learning models. Apart from these machine learning model based on radiomic features, machine learning model based on predictive clinicopathological parameters were also established, which were referred as Cli_Pat model in the study. To assess the contribution of radiomics combined with clinicopathological information to prediction of pCR to NAT for NSCLC, an integrated model named Cli_Pat_Rad_PET/CT was also built to determine its outperformance in contrast with Rad_PET/CT.

### 2.7 Statistical analysis

The IBM SPSS Statistics 27.0.1 and Python 3.7.0 software were employed for statistical analysis. For clinical data, quantitative data that conformed to normal distribution were expressed as mean ± SD, and comparisons between the two groups were conducted using the two independent-sample t-test. Non-normally distributed quantitative information was represented as M (P25–P75), and comparisons between two groups were performed using the Mann–Whitney U test. Qualitative data were compared using either the χ2 test or Fisher’s exact test. Univariate and multivariate logistic regression analyses were performed to select the significant clinicopathological parameters in the prediction of pCR to NAT for NSCLC. A two-sided p-value below 0.05 was considered statistically significant. The prediction results of each model were plotted on a receiver operator characteristic (ROC) curve, and the area under the curve (AUC), sensitivity, specificity, positive predictive value (PPV), and negative predictive value (NPV) were calculated to assess the prediction performance. Thus, a nomogram using logistic regression algorithm involving radiomics and significant clinicopathological indicators was developed to detect its potential application in clinical practice.

## 3 Results

### 3.1 Patient characteristics

A total of 106 patients with NSCLC were eligible and recruited according to inclusion criteria. Based on the pathological outcome to NAT, these included NSCLC patients were classified into two groups: pCR and non-pCR. The differences in the clinicopathologic characteristics between the two groups are presented in **Table 1**. As shown, among all the listed clinicopathologic characteristics, only pathological type (p = 0.012) exhibited statistical significance in distinguishing pCR from non-pCR. With regard to traditional PET metabolic parameters, including SUVmax, SUVpeak, SUVmean, MTV, and TLG, none of them was suggested as potential indicator to predict pathological outcome to NAT for NSCLC.

**Table 1:** Demographic information and clinicopathological characteristics of patients.

Characteristics	Non-PCR (n = 71)	PCR (n = 35)	p-Value
Gender			0.999
Male	62 (87.3%)	31 (88.6%)	
Female	9 (12.7%)	4 (11.4%)	
Age	60.29 ± 8.07	62.57 ± 6.76	0.138
Smoking status			0.708
Current or former	57 (80.3%)	27 (77.1%)	
Never	14 (19.7%)	8 (22.9%)	
BMI	24.89 ± 3.11	25.13 ± 3.15	0.696
NLR	2.37 (1.71–2.99)	2.57 (1.79–3.19)	0.904
PLR	134.71 (104.98–176.10)	150.33 (113.11–182.03)	0.550
Tumor size	4.60 (3.75–6.15)	4.70 (3.05–6.40)	0.407
Tumor location			0.352
Superior lobe of left lung	22 (31.0%)	8 (22.9%)	
Inferior lobe of left lung	11 (15.5%)	4 (11.4%)	
Superior lobe of right lung	15 (21.1%)	14 (40.0%)	
Middle lobe of right lung	7 (9.9%)	2 (5.7%)	
Inferior lobe of right lung	16 (22.5%)	7 (20.0%)	
Pathological type			**0.012** ^*^
Adenocarcinoma	20 (28.2%)	3 (8.6%)	
Squamous carcinoma	46 (64.8%)	32 (91.4%)	
Large cell carcinoma	5 (7.0%)	0 (0.0%)	
Pathological stage			0.449
I	5 (7.0%)	5 (14.3%)	
II	18 (25.4%)	7 (20.0%)	
III	48 (67.6%)	23 (65.7%)	
PD-L1			0.845
Negative	25 (35.2%)	13 (37.1%)	
Positive	46 (64.8%)	22 (62.9%)	
Nodal metastasis			0.707
Negative	27 (38%)	12 (34.3%)	
Positive	44 (62%)	23 (65.7%)	
SUVmax	15.54 (11.90–21.13)	18.58 (12.55–23.04)	0.530
SUVpeak	13.10 (10.02–18.09)	15.97 (10.87–20.35)	0.631
SUVmean	9.31 (7.54–12.54)	11.04 (7.51–14.94)	0.321
MTV	21.91 (12.58–41.66)	19.41 (6.59–38.84)	0.721
TLG	205.26 (114.83–444.61)	220.92 (57.12–550.29)	0.938

A t-test was used for age and BMI; a Mann–Whitney U test was used for NLR, PLR, tumor size, SUVmax, SUVpeak, SUVmean, MTV, and TLG. A χ^2^ test or Fisher’s exact test was used for the rest. ^*^p < 0.05.

BMI, body mass index; NLR, neutrophil-to-lymphocyte ratio; PLR, platelet-to-lymphocyte ratio; PD-L1, programmed death protein ligand 1; SUV, standardized uptake value; MTV, metabolic tumor volume; TLG, total lesion glycolysis.

### 3.2 Radiomics for prediction of pCR to NAT

**Figure 1:**
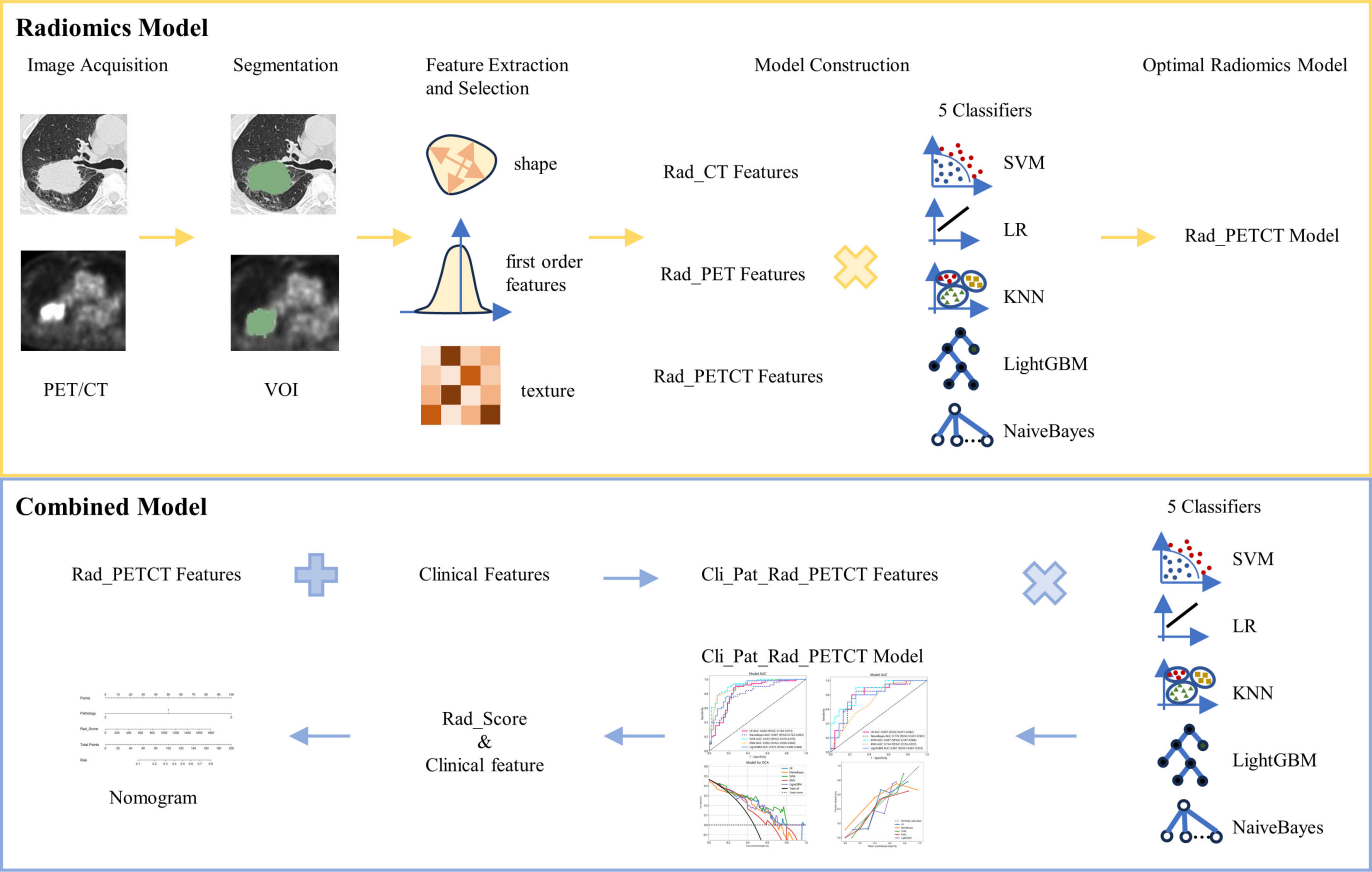
The workflow of radiomic and clinical analysis for image acquisition, segmentation, feature extraction and selection, and model and nomogram construction.

**Figure 2:**
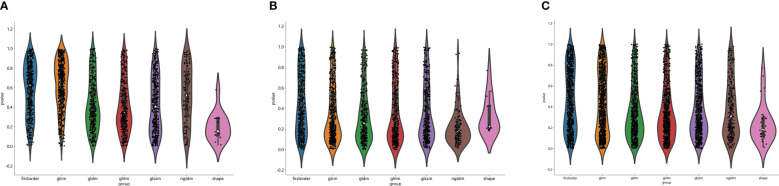
Distribution of radiomic features extracted from CT images only **(A)**, PET images only **(B)** and combined PET/CT images **(C)**, and corresponding p-value results in distinguishing PCR from non-PCR. glcm, gray-level co-occurrence matrix; gldm, gray-level dependence matrix; glrlm, gray-level run length matrix; glszm, gray level size zone matrix; ngtdm, neighborhood gray-tone difference matrix.

**Figure 3:**
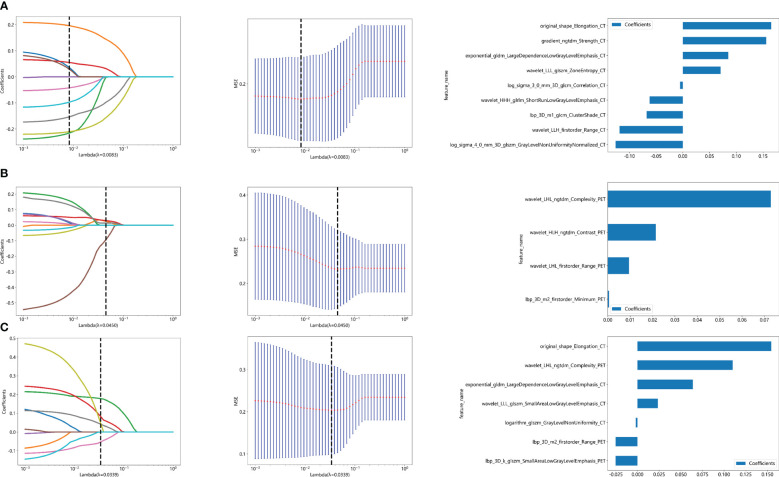
Radiomic feature selection used a LASSO algorithm, which was adjusted by a super parameter (λ), to achieve the purpose of screening the optimal features. The coefficients and mean standard error (MSE) of 10-fold cross validation and the histogram of coefficients of Rad_CT **(A)**, Rad_PET **(B)**, and Rad_PET/CT **(C)** models, respectively. **(A)** The vertical dashed line showed that the corresponding optimal λ value when obtaining the minimum deviation value was λ = 0.0083. Features with non-zero coefficients were screened out corresponding to the vertical lines in the plot, with a total of nine best features selected for Rad_CT model. Correspondingly, **(B)** the optimal λ value was 0.0450, and a total of four optimal features were selected for Rad_PET model. **(C)** The optimal λ value was 0.0339, and a total of seven optimal features were selected for Rad_PET/CT model.

The flow chart of radiomics used in the study to predict pCR to NAT for NSCLC is presented in **Figure 1**. Briefly, the radiomics consist of VOI segmentation, feature extraction, feature selection, model construction and performance evaluation. A total of 4,032 radiomic features (2,016 CT-based and 2,016 PET-based radiomic features) were extracted for each lesion, including morphological, first-order, and texture features. Based on a different combination of selective feature source (PET-based alone radiomics, CT-based alone radiomics, PET/CT-based radiomics, clinicopathological features, and PET/CT radiomics integrated with clinicopathological features) and machine learning classifier (SVM, LR, KNN, LightGBM, and NaiveBayes), 25 machine learning models were finally constructed to select the optimal model for prediction of pCR to NAT. **Figure 2** shows all radiomic features and corresponding p-value results. After radiomic feature extraction and reduction, LASSO regression finally selected nine features for Rad_CT model (**Figure 3A**), four features for Rad_PET model (**Figure 3B**), and seven features for Rad_PET/CT model (**Figure 3C**). Rad_Score was then computed for each patient by combining the selected features linearly, weighted by their respective coefficients. The formulas of Rad_Score for Rad_CT model, Rad_PET model, and Rad_PET/CT model are listed in **Table 2**.

**Table 2:** The establishment of Rad_Score formulas using selected radiomic features based on LASSO algorithm of the three Rad models.

Model Name	Formulas
Rad_CT	Rad_Score = 0.3314405037744722 + 0.070663 × wavelet_LLL_glszm_ZoneEntropy_CT + 0.165537 × original_shape_Elongation_CT - 0.062359 × wavelet_HHH_glrlm_ShortRunLowGrayLevelEmphasis_CT + 0.084869 × exponential_gldm_LargeDependenceLowGrayLevelEmphasis_CT + 0.156102 × gradient_ngtdm_Strength_CT − 0.067547 × lbp_3D_m1_glcm_ClusterShade_CT − 0.118425 × wavelet_LLH_firstorder_Range_CT − 0.125792 × log_sigma_4_0_mm_3D_glszm_GrayLevelNonUniformityNormalized_CT − 0.005096 × log_sigma_3_0_mm_3D_glcm_Correlation_CT
Rad_PET	Rad_Score = 0.3584026346819105 + 0.072922 × wavelet_LHL_ngtdm_Complexity_PET + 0.021449 × wavelet_HLH_ngtdm_Contrast_PET + 0.000527 × lbp_3D_m2_firstorder_Minimum_PET + 0.009493 × wavelet_LHL_firstorder_Range_PET
Rad_PET/CT	Rad_Score = 0.3638561342659228 + 0.154468 × original_shape_Elongation_CT + 0.109735 × wavelet_LHL_ngtdm_Complexity_PET − 0.025411 × lbp_3D_k_glszm_SmallAreaLowGrayLevelEmphasis_PET + 0.023428 × wavelet_LLL_glszm_SmallAreaLowGrayLevelEmphasis_CT − 0.002115 × logarithm_glszm_GrayLevelNonUniformity_CT + 0.063967 × exponential_gldm_LargeDependenceLowGrayLevelEmphasis_CT - 0.025217 × lbp_3D_m2_firstorder_Range_PET

CT, computed tomography; PET, positron emission tomography; PET/CT, positron emission tomography/computed tomography; GLSZM, gray-level size zone matrix; GLRLM, gray-level run length matrix; GLDM, gray-level dependence matrix; GLCM, gray-level co-occurrence matrix.

### 3.3 Machine learning models based on radiomics

**Figure 4:**
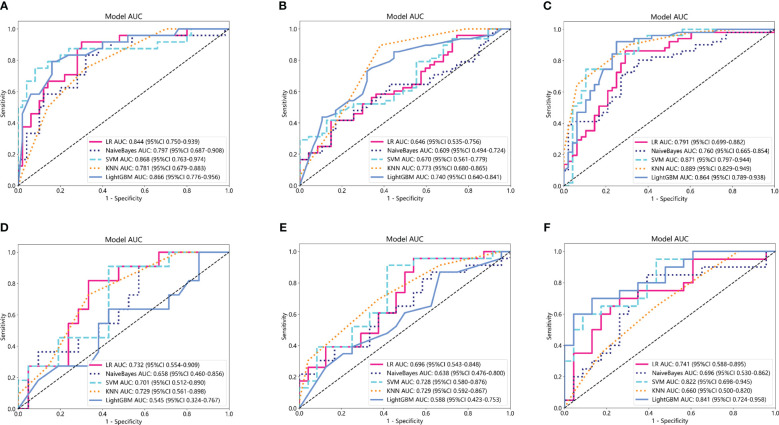
Comparison of ROC curves for the training cohorts of Rad_CT **(A)**, Rad_PET **(B)**, and Rad_PET/CT **(C)** models and testing cohorts of Rad_CT **(D)**, Rad_PET **(E)**, and Rad_PET/CT **(F)** models of five machine learning models. As indicated, the LR-Rad_CT model, KNN-Rad_PET model, and LightGBM-Rad_PET/CT model were the best predicting models, respectively. Among them, the LightGBM-Rad_PET/CT model with an AUC of 0.864 in the training cohort and 0.841 in the testing cohort were considered the optimal model for further analysis.

Based on the aforementioned radiomics model construction after LASSO regression, including Rad_CT model, Rad_PET model, and Rad_PET/CT model, a total of 15 machine learning models were developed by incorporating five different machine learning classifiers (SVM, KNN, LR, LightGBM, and NaiveBayes). ROC analyses were performed to evaluate the performance of these established machine learning models in the prediction of pathological response to NAT for NSCLC using the AUC as the main outcome. As indicated in **Figure 4**, LR-Rad_CT model (**Figures 4A, D**) with an AUC of 0.844 (training cohort) and 0.732 (testing cohort), KNN-Rad_PET model (**Figures 4B, E**) with an AUC of 0.773 (training cohort) and 0.729 (testing cohort) and LightGBM-Rad_PET/CT model (**Figures 4C, F**) with an AUC of 0.864 (training cohort) and 0.841 (testing cohort) were considered the optimal model in Rad_CT models, Rad_PET models, and Rad_PET/CT models, respectively. The decision curves and calibration curves are also depicted and shown in **Supplemenatry Figure S1**. Other measurements, including accuracy, sensitivity, specificity, PPV, and NPV, in training cohort and testing cohort are also calculated and demonstrated in **Supplemenatry Tables S1–S3**.

### 3.4 Machine learning models based on radiomics combined with clinicopathological information

**Figure 5:**
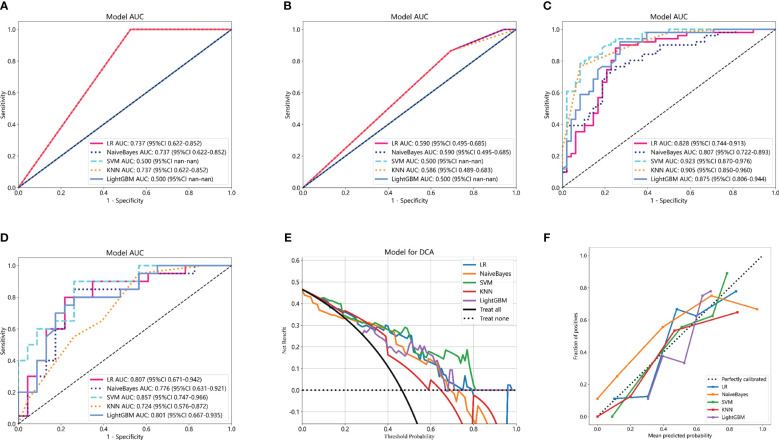
Comparison of ROC curves of five machine learning models for the training cohorts of Cli_Pat **(A)**, testing cohorts of Cli_Pat **(B)**, training cohorts of Cli_Pat_Rad_PET/CT **(C)**, and testing cohorts of Cli_Pat_Rad_PET/CT **(D)** models. The SVM-Cli_Pat_Rad_PET/CT model with an AUC of 0.923 in the training cohort and 0.857 in the testing cohort outperformed other Rad_PET/CT and Cli_Pat_Rad_PET/CT machine learning models. **(E)** The decision curve analysis of the Cli_Pat_Rad_PET/CT models. The SVM-Cli_Pat_Rad_PET/CT model had a higher net benefit in predicting pCR compared to the other four machine learning models. **(F)** The calibration curve of the Cli_Pat_Rad_PET/CT models. The 45° black dashed line represents the ideal prediction performance. The colorful lines of five machine learning models in which closer to the black dashed line represented the higher prediction accuracy. The SVM-Cli_Pat_Rad_PET/CT exhibited the best calibration in predicting pCR status.

Clinicopathological information was reported to potentially provide complementary information to radiomic models. The predictive clinicopathological parameters were selected based on univariate analysis and multivariate analysis. As shown in **Supplemenatry Table S4**, pathological type was found to be significantly related to pCR (p = 0.003), which was also an independent predictor (OR 0.786; 95% CI 0.689–0.897; p = 0.003). In the study, the clinicopathological information was also incorporated with machine learning classifiers to construct Cli_Pat machine learning models (**Figures 5A, B**). Combined Cli_Pat_Rad_PET/CT machine learning models were built by integrating the Rad_PET/CT radiomics with pathological type (**Figures 5C, D**). As indicated in the ROC curves of **Figure 5**, the combined Cli_Pat_Rad_PET/CT machine learning models outperformed the Cli_Pat machine learning models in prediction of pathological response to NAT for NSCLC. Among the five constructed combined Cli_Pat_Rad_PET/CT machine learning models, SVM-Cli_Pat_Rad_PET/CT model outperformed other models with an AUC of 0.923 in the training cohort and an AUC of 0.857 in the testing cohort, which further improved the predictive performance of Rad_PET/CT models. Therefore, the SVM-Cli_Pat_Rad_PET/CT model was selected for the following study. Furthermore, both the decision curve (**Figure 5E**) and the calibration curve (**Figure 5F**) analysis confirmed that the SVM-Cli_Pat_Rad_PET/CT exhibited the highest net benefit and the best calibration in predicting pCR status. Detailed information regarding the performance of all the constructed Cli_Pat machine learning models and Cli_Pat_Rad_PET/CT models were calculated and presented in **Table 3** and **Supplementary Table S5**.

**Table 3:** Each evaluation index of Cli_Pat_Rad_PETCT model in five machine learning algorithms.

Model name	AUC	95% CI	Accuracy	Sensitivity	Specificity	PPV	NPV	
SVM	0.923	0.8704–0.9762	0.894	1.000	0.787	0.825	1.000	Train
SVM	0.857	0.7473–0.9664	0.896	1.000	0.826	0.828	1.000	Test
KNN	0.905	0.8504–0.9603	0.830	0.894	0.766	0.792	0.878	Train
KNN	0.724	0.6762–0.8723	0.792	1.000	0.636	0.706	0.878	Test
LR	0.828	0.7442–0.9131	0.787	0.830	0.745	0.765	0.814	Train
LR	0.807	0.6711–0.9420	0.833	0.958	0.708	0.767	0.944	Test
LightGBM	0.875	0.8062–0.9443	0.819	0.830	0.809	0.812	0.826	Train
LightGBM	0.801	0.6673–0.9354	0.833	0.833	0.870	0.833	0.833	Test
NaiveBayes	0.807	0.7223–0.8934	0.766	0.809	0.723	0.745	0.826	Train
NaiveBayes	0.776	0.6312–0.9213	0.833	0.792	0.875	0.864	0.833	Test

AUC, area under the curve; CI, confidence interval; PPV, positive predictive value; NPV, negative predictive value; SVM, support vector machine; KNN, K-nearest neighbors; LR logistic regression; LightGBM, light gradient boosting machine.

### 3.5 Nomogram construction

**Figure 6:**
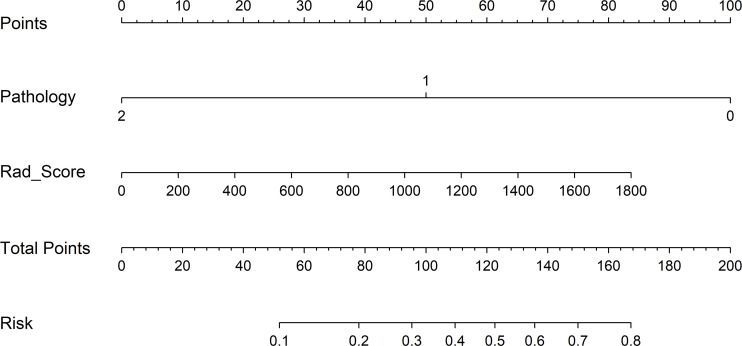
Clinical application of the nomogram in the differentiation of pCR and non-pCR in NSCLC patients. Locate on the pathology and Rad_Score coordinate axis. Calculate and sum the scores corresponding to each point; locate on the total point coordinate axis. The corresponding value on the bottom line is the probability of pathological response to pCR in patients with NSCLC after NAT.

To detect the potential application of the developed PET/CT-derived radiomic models in predicting pCR to NAT for NSCLC, a nomogram using logistic regression algorithm was developed. As shown in **Figure 6**, both Rad_Score and clinicopathological predictor pathological type were involved in nomogram with a visually straightforward representation. In other words, the respective eligible point was endowed to Rad_Score and pathological type based on their different status, and the point for Rad_Score plus the point for pathological type was the total point. Ultimately, the risk of being predicted as pCR to NAT for individual NSCLC was deduced based on the obtained total point.

## 4 Discussion

In the present study, a comprehensive radiomic analysis was performed to determine the role of PET/CT-derived machine learning models in the prediction of pathological response to NAT for NSCLC. To select an optimal model, a total of 25 machine learning models were established based on multiple combinations of different machine learning algorithms and different radiomic feature sources or clinicopathological indicators. Generally, PET/CT-derived radiomic models exhibited improved predictive performance than PET-based alone and CT-based alone radiomic models. The SVM-Rad_PET/CT model outperformed other machine learning models based on radiomic features. Furthermore, the SVM-Cli_Pat_Rad_PET/CT model was finally selected as the optimal model, which enhanced the predictive efficacy of the SVM-Rad_PET/CT model suggesting a complementary role provided by clinicopathological information.

Accurate prediction of pCR to NAT prior treatment was of significance in treatment decision making and survival prognostication for NSCLC (31). Though tremendous efforts were paid to identify potential biomarkers for predicting pCR to NAT, divergences remained to be resolved. Khorrami et al. assessed the role of clinicopathological variables in discerning pathological responses following NAT in NSCLC (25). Among all the variables, only lymphatic invasion exhibited statistical significance in distinguishing (MPR) from non-MPR (OR 0.052; 95% CI 0.007–0.23; p = 0.0006), while none of age, sex, pathological type, vascular infiltration, or tumor volume was found to be without significant differences between the MPR and non-MPR groups. Lin et al. revealed that gender was capable of predicting a good pathological response (GPR) to NAT (p = 0.019) (26). In our study, pathological type was significantly related to pCR (p < 0.05), which was also an independent predictor. The inconsistences in the determination of potential indicators were attributed to different pathological outcome settings and different patient cohorts with various clinical characteristics included in an individual study.

Besides clinical parameters, radiological indicator was also accepted as a non-invasive approach to predict pathological response to NAT for NSCLC. Particularly, several metabolic parameters based on PET/CT images were employed as potential radiological indicator to evaluate the response to NAT. Cui et al. revealed that SUVmax, SUVpeak, and peak SUV corrected for lean body mass (SULpeak) were significantly associated with pCR in patients with stage III NSCLC undergoing neoadjuvant immunochemotherapy therapy followed by surgery (32). Tao et al. discovered a negative correlation between the degree of pathological regression and the SULmax, SULpeak, MTV, and TLG of the preoperative PET/CT (33). Thus, the predictive capability of SUV parameters in determining pathological responses to NAT was verified in several studies for patients with a few types of tumors (34, 35), whereas our study proved that traditional PET/CT parameters (such as SUVmax, SUVmean, SUVpeak, MTV, and TLG) were unable to predict the pathological response to NAT (p > 0.05). Consistent with our results, Antunovic et al. also confirmed that SUVmax and TLG was not suggested as effective predictors of pCR to NAC for breast cancer patients (27). Among these PET metabolic semi-quantitative parameters, choosing a single-pixel value, such as SUV, is not able to comprehensively reflect the intra- and inter-tumoral heterogeneity (36–38).

Radiomics is an emerging hot topic in medical imaging, which is actually a high throughput of feature extraction to reflect the complex heterogeneity existing in the medical images, which is commonly not observed by the naked eye (39–42). Though Lin et al. established a combined radiomic model involving clinical features, radiomic features, and deep learning features for prediction of GPR to immunotherapy-based NAT for NSCLC, there is only a limited amount of radiomic features based on single-mode CT images (26). Despite prior research indicating the improvement of radiomics based on CT in predicting pCR to NAT for NSCLC, few studies involving PET/CT-derived radiomics were currently accessible. While PET/CT, as a dual-modality imaging technique, being capable of providing both anatomical and metabolic information, is expected to improve the performance of CT-based radiomic model in the prediction of pathological response to NAT for NSCLC (43–47). Thus, PET/CT-derived radiomics were increasingly performed to evaluate its power in the prediction of pCR to NAT for NSCLC. As expected, the PET/CT-derived radiomic models were superior to PET-based alone and CT-based alone radiomic models in our study (48, 49). Moreover, five machine learning classifiers were employed and compared to improve the prediction efficiency of the radiomic models. Among the five classifiers used in the present investigation, including LR, SVM, KNN, LightGBM, and NaiveBayes, SVM was found to be the optimal classifier, which was recommended to deal with nonlinear and high-dimensional classification issue with a small-to-medium sample size. Consistently, SVM was proven to be the optimal machine learning algorithm in various studies with respect to radiomics.

Nomogram was commonly used to detect potential application of a model involving multiple predictive indicators in clinical practice (50–52). As indicated in the results, the Cli_Pat_Rad_PET/CT model was proven to be the optimal model with an accuracy of 0.894 in the training cohort and an accuracy of 0.896 in the test cohort, respectively. Thus, a nomogram based on the Cli_Pat_Rad_PET/CT model was depicted in our study. The probability of being predicted as pCR was calculated based on both point of Rad_Score and point of pathological type, which were endowed according to their status. The developed nomogram simplified the procedure of prediction process with a straightforward visualization, which remarkably enhanced its feasibility to be conveniently applied in clinical practice (53, 54). In other words, NSCLC patients with a higher probability of being predicted as pCR to NAT, intensive administration is expected to maximize the therapeutic efficacy. In contrast, for NSCLC patients with a lower probability of being predicted as pCR to NAT, modified NAT or other effective treatment choice should be suggested in early stage of therapy process. Accurate prediction of pCR to NAT for NSCLC before treatment significantly contributing to therapeutic decision making promisingly improves the clinical outcome of NSCLC.

Although promising findings were obtained in this study, several limitations remained to be addressed. First, this was a retrospective study with a relatively small sample size, which was performed in one institution. A multi-center prospective study with an adequate sample size is warranted in the future to further verify the conclusion. Then, a subgroup analysis according to the type of NAT was not conducted due to the limited number of included NSCLC patients. Therefore, the status of PD-L1 was not involved to construct the combined machine learning model with clinicopathological information in the study because PD-L1 was usually assessed for NSCLC with immune checkpoint inhibitor treatment-based NAT. In the end, to improve the performance of artificial intelligence models based on radiological images in the prediction of pathological response to NAT, deep learning models involving VOI of both tumor lesion itself and peri-tumor region are considered a promising choice.

## 5 Conclusion

Machine learning models constructed based on PET/CT-derived radiomics were able to effectively predict pathological response to NAT prior treatment for NSCLC, and their predictive performances were further enhanced by the developed combined model involving PET/CT-derived radiomics and clinicopathological information. Therefore, the SVM-Cli_Pat_Rad_PET/CT model was potentially used a non-invasive tool to optimize personalized treatment and improve the clinical prospect of NAT for NSCLC.

## Data availability statement

The original contributions presented in the study are included in the article/**Supplementary Material**. Further inquiries can be directed to the corresponding authors.

## Ethics statement

The studies involving humans were approved by Ethics Review Committee of Tianjin Medical University Cancer Institute and Hospital. The studies were conducted in accordance with the local legislation and institutional requirements. The written informed consent from all enrolled patients was waived with the confirmation of patient data confidentiality in the retrospective study.

## Author contributions

JL: Writing – original draft, Methodology, Funding acquisition, Formal analysis, Data curation, Conceptualization. CS: Writing – original draft, Investigation, Formal analysis, Data curation, Conceptualization. HB: Writing – original draft, Methodology, Formal analysis, Data curation. YL: Writing – original draft, Data curation. ZW: Writing – original draft, Data curation. JF: Writing – original draft, Data curation. LQ: Writing – original draft, Resources, Data curation. KC: Writing – original draft, Data curation. WX: Writing – review & editing, Supervision, Resources. XL: Writing – review & editing, Supervision, Funding acquisition, Conceptualization.

## References

[1] Siegel RL Miller KD Wagle NS Jemal A. Cancer statistics, 2023. CA Cancer J Clin. (2023) 73:17–48. doi: 10.3322/caac.21763 36633525

[2] Monaco L De Bernardi E Bono F Cortinovis D Crivellaro C Elisei F. The "digital biopsy" in non-small cell lung cancer (NSCLC): a pilot study to predict the PD-L1 status from radiomics features of [18F]FDG PET/CT. Eur J Nucl Med Mol Imaging. (2022) 49:3401–11. doi: 10.1007/s00259-022-05783-z 35403860

[3] Yuan Z Yu X Wu S Wu X Wang Q Cheng W. Instability mechanism of osimertinib in plasma and a solving strategy in the pharmacokinetics study. Front Pharmacol. (2022) 13:928983. doi: 10.3389/fphar.2022.928983 35935836 PMC9354582

[4] Yoo J Lee J Cheon M Kim H Choi YS Pyo H. Radiomics analysis of (18)F-FDG PET/CT for prognosis prediction in patients with stage III non-small cell lung cancer undergoing neoadjuvant chemoradiation therapy followed by surgery. Cancers (Basel). (2023) 15:2012. doi: 10.3390/cancers15072012 37046673 PMC10093358

[5] Saw SPL Ong BH Chua KLM Takano A Tan DSW. Revisiting neoadjuvant therapy in non-small-cell lung cancer. Lancet Oncol. (2021) 22:e501–e16. doi: 10.1016/S1470-2045(21)00383-1 34735819

[6] Liu C Zhao W Xie J Lin H Hu X Li C. Development and validation of a radiomics-based nomogram for predicting a major pathological response to neoadjuvant immunochemotherapy for patients with potentially resectable non-small cell lung cancer. Front Immunol. (2023) 14:1115291. doi: 10.3389/fimmu.2023.1115291 36875128 PMC9978193

[7] Beukinga RJ Hulshoff JB Mul VEM Noordzij W Kats-Ugurlu G Slart R. Prediction of response to neoadjuvant chemotherapy and radiation therapy with baseline and restaging (18)F-FDG PET imaging biomarkers in patients with esophageal cancer. Radiology. (2018) 287:983–92. doi: 10.1148/radiol.2018172229 29533721

[8] Chen K Wang J Li S Zhou W Xu W. Predictive value of 18F-FDG PET/CT-based radiomics model for neoadjuvant chemotherapy efficacy in breast cancer: a multi-scanner/center study with external validation. Eur J Nucl Med Mol Imaging. (2023) 50:1869–80. doi: 10.1007/s00259-023-06150-2 36808002

[9] Li P Wang X Xu C Liu C Zheng C Fulham MJ. (18)F-FDG PET/CT radiomic predictors of pathologic complete response (pCR) to neoadjuvant chemotherapy in breast cancer patients. Eur J Nucl Med Mol Imaging. (2020) 47:1116–26. doi: 10.1007/s00259-020-04684-3 31982990

[10] Umutlu L Kirchner J Bruckmann NM Morawitz J Antoch G Ting S. Multiparametric (18)F-FDG PET/MRI-based radiomics for prediction of pathological complete response to neoadjuvant chemotherapy in breast cancer. Cancers (Basel). (2022) 14:1727. doi: 10.3390/cancers14071727 35406499 PMC8996836

[11] Yin X Liao H Yun H Lin N Li S Xiang Y. Artificial intelligence-based prediction of clinical outcome in immunotherapy and targeted therapy of lung cancer. Semin Cancer Biol. (2022) 86:146–59. doi: 10.1016/j.semcancer.2022.08.002 35963564

[12] Dercle L Sun S Seban R-D Mekki A Sun R Tselikas L. Emerging and evolving concepts in cancer immunotherapy imaging. Radiology. (2023) 306:32–46. doi: 10.1148/radiol.210518 36472538

[13] Chen L Liu K Zhao X Shen H Zhao K Zhu W. Habitat imaging-based (18)F-FDG PET/CT radiomics for the preoperative discrimination of non-small cell lung cancer and benign inflammatory diseases. Front Oncol. (2021) 11:759897. doi: 10.3389/fonc.2021.759897 34692548 PMC8526895

[14] Fiz F Masci C Costa G Sollini M Chiti A Ieva F. PET/CT-based radiomics of mass-forming intrahepatic cholangiocarcinoma improves prediction of pathology data and survival. Eur J Nucl Med Mol Imaging. (2022) 49:3387–400. doi: 10.1007/s00259-022-05765-1 35347437

[15] Hannequin P Decroisette C Kermanach P Berardi G Bourbonne V. FDG PET and CT radiomics in diagnosis and prognosis of non-small-cell lung cancer. Transl Lung Cancer Res. (2022) 11:2051–63. doi: 10.21037/tlcr-22-158 PMC9641045 36386457

[16] Li J Ge S Sang S Hu C Deng S. Evaluation of PD-L1 expression level in patients with non-small cell lung cancer by (18)F-FDG PET/CT radiomics and clinicopathological characteristics. Front Oncol. (2021) 11:789014. doi: 10.3389/fonc.2021.789014 34976829 PMC8716940

[17] Zhou J Zou S Kuang D Yan J Zhao J Zhu X. A novel approach using FDG-PET/CT-based radiomics to assess tumor immune phenotypes in patients with non-small cell lung cancer. Front Oncol. (2021) 11:769272. doi: 10.3389/fonc.2021.769272 34868999 PMC8635743

[18] Orlhac F Boughdad S Philippe C Stalla-Bourdillon H Nioche C Champion L. A postreconstruction harmonization method for multicenter radiomic studies in PET. J Nucl Med. (2018) 59:1321–8. doi: 10.2967/jnumed.117.199935 29301932

[19] Mazzaschi G Milanese G Pagano P Madeddu D Gnetti L Trentini F. Integrated CT imaging and tissue immune features disclose a radio-immune signature with high prognostic impact on surgically resected NSCLC. Lung Cancer. (2020) 144:30–9. doi: 10.1016/j.lungcan.2020.04.006 32361033

[20] Wang X Yang W Zhou Q Luo H Chen W Yeung SJ. The role of (18)F-FDG PET/CT in predicting the pathological response to neoadjuvant PD-1 blockade in combination with chemotherapy for resectable esophageal squamous cell carcinoma. Eur J Nucl Med Mol Imaging. (2022) 49:4241–51. doi: 10.1007/s00259-022-05872-z 35732974

[21] Murakami Y Kawahara D Tani S Kubo K Katsuta T Imano N. Predicting the local response of esophageal squamous cell carcinoma to neoadjuvant chemoradiotherapy by radiomics with a machine learning method using (18)F-FDG PET images. Diagnostics (Basel). (2021) 11:1049. doi: 10.3390/diagnostics11061049 34200332 PMC8227132

[22] Mu W Jiang L Shi Y Tunali I Gray JE Katsoulakis E. Non-invasive measurement of PD-L1 status and prediction of immunotherapy response using deep learning of PET/CT images. J Immunother Cancer. (2021) 9:e002118. doi: 10.1136/jitc-2020-002118 34135101 PMC8211060

[23] Jimenez JE Abdelhafez A Mittendorf EA Elshafeey N Yung JP Litton JK. A model combining pretreatment MRI radiomic features and tumor-infiltrating lymphocytes to predict response to neoadjuvant systemic therapy in triple-negative breast cancer. Eur J Radiol. (2022) 149:110220. doi: 10.1016/j.ejrad.2022.110220 35193025

[24] Ninatti G Kirienko M Neri E Sollini M Chiti A. Imaging-based prediction of molecular therapy targets in NSCLC by radiogenomics and AI approaches: A systematic review. Diagnostics. (2020) 10:359. doi: 10.3390/diagnostics10060359 32486314 PMC7345054

[25] Khorrami M Jain P Bera K Alilou M Thawani R Patil P. Predicting pathologic response to neoadjuvant chemoradiation in resectable stage III non-small cell lung cancer patients using computed tomography radiomic features. Lung Cancer. (2019) 135:1–9. doi: 10.1016/j.lungcan.2019.06.020 31446979 PMC6711393

[26] Lin Q Wu HJ Song QS Tang YK. CT-based radiomics in predicting pathological response in non-small cell lung cancer patients receiving neoadjuvant immunotherapy. Front Oncol. (2022) 12:937277. doi: 10.3389/fonc.2022.937277 36267975 PMC9577189

[27] Antunovic L De Sanctis R Cozzi L Kirienko M Sagona A Torrisi R. PET/CT radiomics in breast cancer: promising tool for prediction of pathological response to neoadjuvant chemotherapy. Eur J Nucl Med Mol Imaging. (2019) 46:1468–77. doi: 10.1007/s00259-019-04313-8 30915523

[28] Tong H Sun J Fang J Zhang M Liu H Xia R. A machine learning model based on PET/CT radiomics and clinical characteristics predicts tumor immune profiles in non-small cell lung cancer: A retrospective multicohort study. Front Immunol. (2022) 13:859323. doi: 10.3389/fimmu.2022.859323 35572597 PMC9105942

[29] Zhao X Zhao Y Zhang J Zhang Z Liu L Zhao X. Predicting PD-L1 expression status in patients with non-small cell lung cancer using [18F]FDG PET/CT radiomics. EJNMMI Res. (2023) 13:4. doi: 10.1186/s13550-023-00956-9 36682020 PMC9868196

[30] Xie D Xu F Zhu W Pu C Huang S Lou K. Delta radiomics model for the prediction of progression-free survival time in advanced non-small-cell lung cancer patients after immunotherapy. Front Oncol. (2022) 12:990608. doi: 10.3389/fonc.2022.990608 36276082 PMC9583844

[31] Wang C Ma J Shao J Zhang S Liu Z Yu Y. Predicting EGFR and PD-L1 status in NSCLC patients using multitask AI system based on CT images. Front Immunol. (2022) 13:813072. doi: 10.3389/fimmu.2022.813072 35250988 PMC8895233

[32] Cui Y Lin Y Zhao Z Long H Zheng L Lin X. Comprehensive (18)F-FDG PET-based radiomics in elevating the pathological response to neoadjuvant immunochemotherapy for resectable stage III non-small-cell lung cancer: A pilot study. Front Immunol. (2022) 13:994917. doi: 10.3389/fimmu.2022.994917 36466929 PMC9713843

[33] Tao X Li N Wu N He J Ying J Gao S. The efficiency of (18)F-FDG PET-CT for predicting the major pathologic response to the neoadjuvant PD-1 blockade in resectable non-small cell lung cancer. Eur J Nucl Med Mol Imaging. (2020) 47:1209–19. doi: 10.1007/s00259-020-04711-3 PMC7101299 32043180

[34] Yang L Chang J He X Peng M Zhang Y Wu T. PET/CT-based radiomics analysis may help to predict neoadjuvant chemotherapy outcomes in breast cancer. Front Oncol. (2022) 12:849626. doi: 10.3389/fonc.2022.849626 36419895 PMC9676961

[35] Giannini V Mazzetti S Bertotto I Chiarenza C Cauda S Delmastro E. Predicting locally advanced rectal cancer response to neoadjuvant therapy with (18)F-FDG PET and MRI radiomics features. Eur J Nucl Med Mol Imaging. (2019) 46:878–88. doi: 10.1007/s00259-018-4250-6 30637502

[36] Polverari G Ceci F Bertaglia V Reale ML Rampado O Gallio E. 18F-FDG pet parameters and radiomics features analysis in advanced nsclc treated with immunotherapy as predictors of therapy response and survival. Cancers. (2020) 12:1163. doi: 10.3390/cancers12051163 32380754 PMC7281558

[37] Wang X Xu C Grzegorzek M Sun H. Habitat radiomics analysis of pet/ct imaging in high-grade serous ovarian cancer: Application to Ki-67 status and progression-free survival. Front Physiol. (2022) 13:948767. doi: 10.3389/fphys.2022.948767 36091379 PMC9452776

[38] Beaumont J Acosta O Devillers A Palard-Novello X Chajon E de Crevoisier R. Voxel-based identification of local recurrence sub-regions from pre-treatment PET/CT for locally advanced head and neck cancers. EJNMMI Res. (2019) 9:90. doi: 10.1186/s13550-019-0556-z 31535233 PMC6751236

[39] Wang C Xu X Shao J Zhou K Zhao K He Y. Deep learning to predict EGFR mutation and PD-L1 expression status in non-small-cell lung cancer on computed tomography images. J Oncol. (2021) 2021:5499385. doi: 10.1155/2021/5499385 35003258 PMC8741343

[40] Wang C Ma J Shao J Zhang S Li J Yan J. Non-invasive measurement using deep learning algorithm based on multi-source features fusion to predict PD-L1 expression and survival in NSCLC. Front Immunol. (2022) 13:828560. doi: 10.3389/fimmu.2022.828560 35464416 PMC9022118

[41] Napel S Mu W Jardim-Perassi BV Aerts H Gillies RJ. Quantitative imaging of cancer in the postgenomic era: Radio(geno)mics, deep learning, and habitats. Cancer. (2018) 124:4633–49. doi: 10.1002/cncr.31630 PMC6482447 30383900

[42] Sala E Mema E Himoto Y Veeraraghavan H Brenton JD Snyder A. Unravelling tumour heterogeneity using next-generation imaging: radiomics, radiogenomics, and habitat imaging. Clin Radiol. (2017) 72:3–10. doi: 10.1016/j.crad.2016.09.013 27742105 PMC5503113

[43] Leger MA Routy B Juneau D. FDG PET/CT for evaluation of immunotherapy response in lung cancer patients. Semin Nucl Med. (2022) 52:707–19. doi: 10.1053/j.semnuclmed.2022.04.010 35636978

[44] Ding E Lu D Wei L Feng X Shen J Xu W. Predicting tumor recurrence using metabolic indices of (18)F-FDG PET/CT prior to orthotopic liver transplantationfor hepatocellular carcinoma. Oncol Lett. (2020) 20:1245–55. doi: 10.3892/ol.2020.11681 PMC7377045 32724365

[45] Leithner D Schoder H Haug A Vargas HA Gibbs P Haggstrom I. Impact of comBat harmonization on PET radiomics-based tissue classification: A dual-center PET/MRI and PET/CT study. J Nucl Med. (2022) 63:1611–6. doi: 10.2967/jnumed.121.263102 PMC9536705 35210300

[46] Li Y Zhang Y Fang Q Zhang X Hou P Wu H. Radiomics analysis of [(18)F]FDG PET/CT for microvascular invasion and prognosis prediction in very-early- and early-stage hepatocellular carcinoma. Eur J Nucl Med Mol Imaging. (2021) 48:2599–614. doi: 10.1007/s00259-020-05119-9 33416951

[47] Evangelista L Cuocolo A Pace L Mansi L Del Vecchio S Miletto P. Performance of FDG-PET/CT in solitary pulmonary nodule based on pre-test likelihood of Malignancy: results from the ITALIAN retrospective multicenter trial. Eur J Nucl Med Mol Imaging. (2018) 45:1898–907. doi: 10.1007/s00259-018-4016-1 29736699

[48] Chen Y Wang Z Yin G Sui C Liu Z Li X. Prediction of HER2 expression in breast cancer by combining PET/CT radiomic analysis and machine learning. Ann Nucl Med. (2022) 36:172–82. doi: 10.1007/s12149-021-01688-3 34716873

[49] Tixier F Cheze-le-Rest C Schick U Simon B Dufour X Key S. Transcriptomics in cancer revealed by Positron Emission Tomography radiomics. Sci Rep. (2020) 10:5660. doi: 10.1038/s41598-020-62414-z 32221360 PMC7101432

[50] Wang X Xie T Luo J Zhou Z Yu X Guo X. Radiomics predicts the prognosis of patients with locally advanced breast cancer by reflecting the heterogeneity of tumor cells and the tumor microenvironment. Breast Cancer Res. (2022) 24:20. doi: 10.1186/s13058-022-01516-0 35292076 PMC8922933

[51] Cui L Yu T Kan Y Dong Y Luo Y Jiang X. Multi-parametric MRI-based peritumoral radiomics on prediction of lymph-vascular space invasion in early-stage cervical cancer. Diagn Interv Radiol. (2022) 28:312–21. doi: 10.5152/dir.2022.20657 PMC9634933 35731710

[52] Mu W Liang Y Hall LO Tan Y Balagurunathan Y Wenham R. (18)F-FDG PET/CT habitat radiomics predicts outcome of patients with cervical cancer treated with chemoradiotherapy. Radiol Artif Intell. (2020) 2:e190218. doi: 10.1148/ryai.2020190218 33937845 PMC8082355

[53] Wang Y Luo S Jin G Fu R Yu Z Zhang J. Preoperative clinical-radiomics nomogram for microvascular invasion prediction in hepatocellular carcinoma using [Formula: see text]F-FDG PET/CT. BMC Med Imaging. (2022) 22:70. doi: 10.1186/s12880-022-00796-4 35428272 PMC9013080

[54] Wang Q Li C Zhang J Hu X Fan Y Ma K. Radiomics models for predicting microvascular invasion in hepatocellular carcinoma: A systematic review and radiomics quality score assessment. Cancers (Basel). (2021) 13:5864. doi: 10.3390/cancers13225864 34831018 PMC8616379

