# Supplementary material for: Radiomics based on 18F-FDG PET/CT for prediction of pathological complete response to neoadjuvant therapy in non-small cell lung cancer
Source: Front Oncol. 2024 Jul 26;14:1425837. doi: 10.3389/fonc.2024.1425837 (PMC11310012; doi:10.3389/fonc.2024.1425837)
Supplement: Supplementary file 1 [file DataSheet_1.doc]

**Supplementary Material**

^18^F-FDG PET/CT-Based Radiomics for Prediction of Pathological Complete Response to Neoadjuvant Therapy in Non-small Cell Lung Cancer

**Materials and Methods**

**Study population**

The inclusion criteria and exclusion criteria for this study were listed as follows: Inclusion Criteria : (i) pathologic biopsy confirmed NSCLC; (ii) ^18^F-FDG PET/CT prior to any treatment; (iii) administration of neoadjuvant chemotherapy with or without immunotherapy.

Exclusion criteria: (i) multifocal NSCLC (n=14); (ii) simultaneous occurrence of multiple types of tumors (n=9); (iii) lack of complete NAT regimen (n=48); (iv) suboptimal image quality in PET/CT examinations (n=11).

**Image segmentation and feature extraction**

The obtained quantitative radiomic features were classified into three distinct groups: morphological features (n=14), grayscale statistic features (n=396) and texture features (n=1606). In detail, morphological features (n=14) depicted the three-dimensional shape characteristics. Grayscale statistic features (n=396) illustrated the first-order statistical distribution of voxel intensities within the VOI. Texture features (n=1606) outlined the patterns, as well as the second- and high-order spatial distributions of the voxel intensities within the VOI. The texture features consisted of the gray-level co-occurrence matrix (GLCM) (n=484), gray-level dependence matrix (GLDM) (n=308), gray-level run length matrix (GLRLM) (n=352), gray level size zone matrix (GLSZM) (n=352) and neighborhood gray-tone difference matrix (NGTDM) (n=110) in the study.


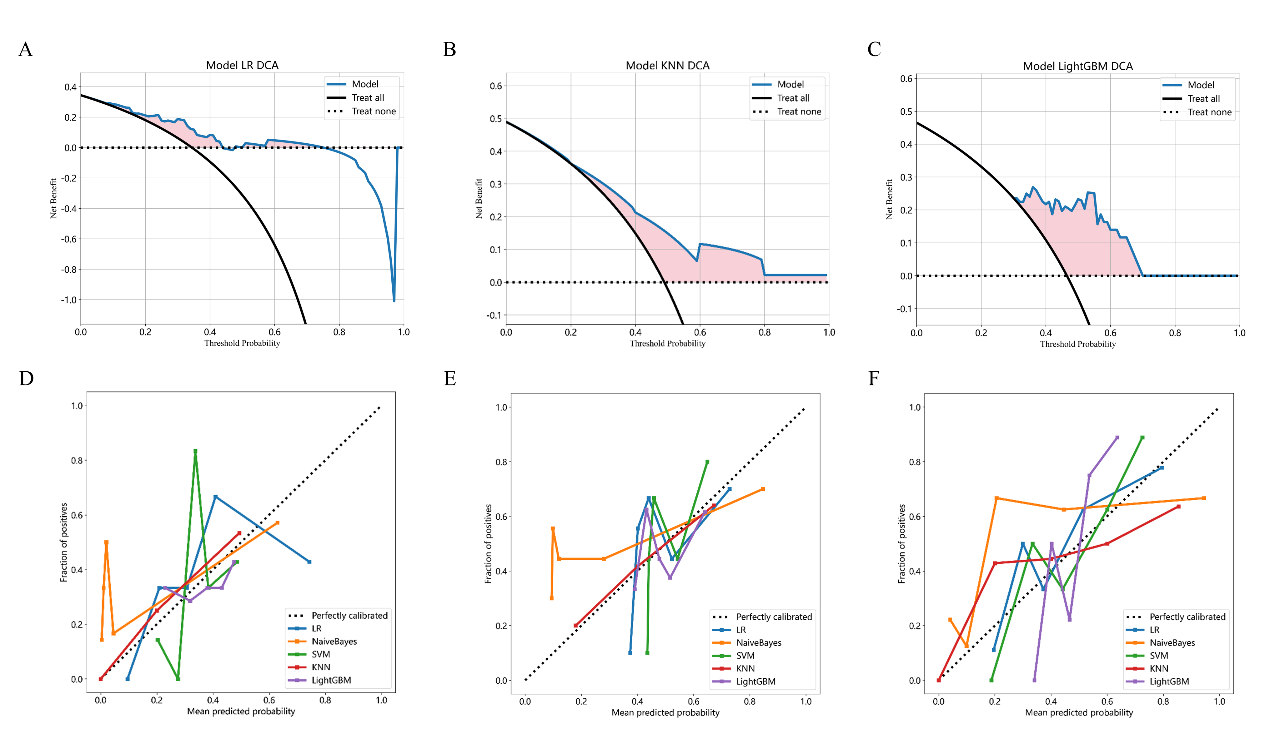


**Fig S1** The decision curve analysis of the best machine learning models in Rad_CT (**A**), Rad_PET (**B**), and Rad_PETCT (**C**) models, and the calibration curves of the five machine learning models in Rad_CT (**D**), Rad_PET (**E**), and Rad_PETCT (**F**) models respectively.

**Table S1** Each evaluation index of Rad_CT model in five machine learning algorithms

| **Model Name** | **AUC** | **95%CI** | **Accuracy** | **Sensitivity** | **Specificity** | **PPV** | **NPV** |  |
| --- | --- | --- | --- | --- | --- | --- | --- | --- |
| SVM | 0.868 | 0.7630-0.9737 | 0.865 | 0.750 | 0.920 | 0.818 | 0.885 | train |
| SVM | 0.701 | 0.5121-0.8905 | 0.688 | 0.909 | 0.571 | 0.526 | 0.923 | test |
| KNN | 0.781 | 0.6792-0.8825 | 0.703 | 0.750 | 0.694 | 0.529 | 0.850 | train |
| KNN | 0.729 | 0.5605-0.8984 | 0.688 | 0.727 | 0.667 | 0.533 | 0.824 | test |
| LR | 0.844 | 0.7496-0.9387 | 0.770 | 0.917 | 0.700 | 0.595 | 0.946 | train |
| LR | 0.732 | 0.5541-0.9092 | 0.719 | 0.818 | 0.700 | 0.562 | 0.875 | test |
| LightGBM | 0.866 | 0.7764-0.9560 | 0.824 | 0.792 | 0.857 | 0.704 | 0.894 | train |
| LightGBM | 0.545 | 0.3241-0.7668 | 0.594 | 0.636 | 0.600 | 0.437 | 0.750 | test |
| NaiveBayes | 0.798 | 0.6868-0.9082 | 0.730 | 0.833 | 0.680 | 0.556 | 0.895 | train |
| NaiveBayes | 0.658 | 0.4597-0.8564 | 0.594 | 0.909 | 0.450 | 0.455 | 0.900 | test |

**Table S2** Each evaluation index of Rad_PET model in five machine learning algorithms

| **Model Name** | **AUC** | **95%CI** | **Accuracy** | **Sensitivity** | **Specificity** | **PPV** | **NPV** |  |
| --- | --- | --- | --- | --- | --- | --- | --- | --- |
| SVM | 0.670 | 0.5613-0.7787 | 0.642 | 0.292 | 1.000 | 1.000 | 1.000 | train |
| SVM | 0.728 | 0.5802-0.8763 | 0.745 | 0.913 | 0.583 | 0.677 | 0.875 | test |
| KNN | 0.773 | 0.6804-0.8648 | 0.758 | 0.896 | 0.617 | 0.705 | 0.853 | train |
| KNN | 0.729 | 0.5918-0.8665 | 0.660 | 0.696 | 0.625 | 0.640 | 0.682 | test |
| LR | 0.646 | 0.5353-0.7564 | 0.632 | 0.417 | 0.851 | 0.741 | 0.588 | train |
| LR | 0.696 | 0.5431-0.8482 | 0.702 | 0.957 | 0.458 | 0.629 | 0.917 | test |
| LightGBM | 0.740 | 0.6400-0.8410 | 0.705 | 0.729 | 0.727 | 0.700 | 0.711 | train |
| LightGBM | 0.588 | 0.4231-0.7526 | 0.596 | 0.870 | 0.381 | 0.556 | 0.727 | test |
| NaiveBayes | 0.609 | 0.4936-0.7236 | 0.621 | 0.417 | 0.830 | 0.714 | 0.582 | train |
| NaiveBayes | 0.638 | 0.4758-0.7996 | 0.617 | 0.870 | 0.375 | 0.571 | 0.750 | test |

**Table S3** Each evaluation index of Rad_PETCT model in five machine learning algorithms

| **Model Name** | **AUC** | **95%CI** | **Accuracy** | **Sensitivity** | **Specificity** | **PPV** | **NPV** |  |
| --- | --- | --- | --- | --- | --- | --- | --- | --- |
| SVM | 0.871 | 0.7970-0.9440 | 0.818 | 0.745 | 0.915 | 0.884 | 0.768 | train |
| SVM | 0.822 | 0.6985-0.9450 | 0.744 | 0.950 | 0.565 | 0.655 | 0.929 | test |
| KNN | 0.889 | 0.8292-0.9486 | 0.798 | 0.902 | 0.688 | 0.754 | 0.868 | train |
| KNN | 0.660 | 0.4998-0.8197 | 0.605 | 0.650 | 0.591 | 0.565 | 0.650 | test |
| LR | 0.791 | 0.6995-0.8822 | 0.788 | 0.863 | 0.708 | 0.759 | 0.829 | train |
| LR | 0.741 | 0.5876-0.8950 | 0.721 | 0.700 | 0.739 | 0.700 | 0.739 | test |
| LightGBM | 0.864 | 0.7894-0.9378 | 0.838 | 0.922 | 0.750 | 0.797 | 0.900 | train |
| LightGBM | 0.841 | 0.7243-0.9583 | 0.791 | 0.700 | 0.870 | 0.824 | 0.769 | test |
| NaiveBayes | 0.760 | 0.6654-0.8543 | 0.727 | 0.804 | 0.646 | 0.707 | 0.756 | train |
| NaiveBayes | 0.696 | 0.5297-0.8616 | 0.721 | 0.850 | 0.609 | 0.654 | 0.824 | test |

**Table S4** Univariate regression analysis and multivariate regression analysis in predicting pCR

|  | **Univariate** | | | **Multivariate** | | |
| --- | --- | --- | --- | --- | --- | --- |
| **Characteristics** | **OR** | **95%CI** | **P value** | **OR** | **95%CI** | **P value** |
| Gender | 1.026 | 0.812-1.296 | 0.856 |  |  |  |
| Age | 1.008 | 0.998-1.017 | 0.186 |  |  |  |
| Smoking status | 0.959 | 0.794-1.157 | 0.711 |  |  |  |
| BMI | 1.002 | 0.977-1.026 | 0.916 |  |  |  |
| NLR | 1.035 | 0.967-1.107 | 0.404 |  |  |  |
| PLR | 1.001 | 1.000-1.002 | 0.284 |  |  |  |
| Tumor size | 0.972 | 0.934-1.011 | 0.231 |  |  |  |
| Tumor location | 0.941 | 0.892-0.992 | 0.059 |  |  |  |
| Pathological type | 0.786 | 0.689-0.897 | **0.003**^**^ | 0.786 | 0.689-0.897 | **0.003**^**^ |
| Pathological stage | 0.973 | 0.873-1.083 | 0.673 |  |  |  |
| PDL1 | 0.982 | 0.837-1.151 | 0.847 |  |  |  |
| Nodal metastasis | 1.036 | 0.884-1.214 | 0.710 |  |  |  |
| SUVmax | 1.002 | 0.993-1.011 | 0.749 |  |  |  |
| SUVpeak | 1.001 | 0.991-1.011 | 0.871 |  |  |  |
| SUVmean | 1.006 | 0.992-1.019 | 0.487 |  |  |  |
| MTV | 0.999 | 0.997-1.001 | 0.528 |  |  |  |
| TLG | 1.193 | 0.799-1.781 | 0.921 |  |  |  |

OR, odds ratio; CI, confidence interval

^**^ *p*<0.01

**Table S5** Each evaluation index of Cli_Pat model in five machine learning algorithms

| **Model Name** | **AUC** | **95%CI** | **Accuracy** | **Sensitivity** | **Specificity** | **PPV** | **NPV** |  |
| --- | --- | --- | --- | --- | --- | --- | --- | --- |
| SVM | 0.500 | 1.0000-1.0000 | 0.297 | 1.000 | - | 0.297 | 0.000 | train |
| SVM | 0.500 | 1.0000-1.0000 | 0.406 | 1.000 | - | 0.406 | 0.000 | test |
| KNN | 0.586 | 0.4887-0.6826 | 0.473 | 0.864 | 1.0 | 0.345 | 0.842 | train |
| KNN | 0.737 | 0.6215-0.8522 | 0.688 | 1.000 | 1.0 | 0.565 | 1.000 | test |
| LR | 0.590 | 0.4946-0.6846 | 0.473 | 0.864 | 1.0 | 0.345 | 0.842 | train |
| LR | 0.688 | 0.6215-0.8522 | 0.688 | 1.000 | 1.0 | 0.565 | 1.000 | test |
| LightGBM | 0.500 | 1.0000-1.0000 | 0.297 | 1.000 | - | 0.297 | 0.000 | train |
| LightGBM | 0.500 | 1.0000-1.0000 | 0.406 | 1.000 | - | 0.406 | 0.000 | test |
| NaiveBayes | 0.590 | 0.4946-0.6846 | 0.473 | 0.864 | 1.0 | 0.345 | 0.842 | train |
| NaiveBayes | 0.737 | 0.6215-0.8522 | 0.688 | 1.000 | 1.0 | 0.565 | 1.000 | test |
